# The Proteasome Inhibitor Bortezomib Affects Chondrosarcoma Cells via the Mitochondria-Caspase Dependent Pathway and Enhances Death Receptor Expression and Autophagy

**DOI:** 10.1371/journal.pone.0168193

**Published:** 2016-12-15

**Authors:** Birgit Lohberger, Bibiane Steinecker-Frohnwieser, Nicole Stuendl, Heike Kaltenegger, Andreas Leithner, Beate Rinner

**Affiliations:** 1 Department of Orthopedic Surgery, Medical University Graz, Graz, Austria; 2 Ludwig Boltzmann Institute for Rehabilitation of Internal Diseases, Ludwig Boltzmann Cluster for Rheumatology, Balneology and Rehabilitation, Saalfelden, Austria; 3 Institute of Biophysics, Medical University of Graz, Graz, Austria; 4 Division of Biomedical Research, Medical University of Graz, Graz, Austria; Univerzitet u Beogradu, SERBIA

## Abstract

High grade chondrosarcoma is characterized by its lack of response to conventional cytotoxic chemotherapy, the tendency to develop lung metastases, and low survival rates. Research within the field prioritizes the development and expansion of new treatment options for dealing with unresectable or metastatic diseases. Numerous clinical trials using the proteasome inhibitor bortezomib have shown specific efficacy as an active antitumor agent for treating a variety of solid tumors. However, as of yet the effect of bortezomib on chondrosarcoma has not been investigated. In our study, bortezomib decreased cell viability and proliferation in two different chondrosarcoma cell lines in a time- and dose dependent manner. FACS analysis, mRNA- and protein expression studies illustrated that induction of apoptosis developed through the intrinsic mitochondria-caspase dependent pathway. Furthermore, bortezomib treatment significantly increased expression of the death receptors TRAILR-1 and TRAILR-2 in chondrosarcoma cells. An increased expression of the autophagy markers Atg5/12, Beclin, and LC3BI-II supports the interpretation that bortezomib functions as a trigger for autophagy. Our results demonstrated for the first time that bortezomib reduced viability and proliferation of chondrosarcoma cells, induced apoptosis via the mitochondria-caspase dependent pathway and enhanced death receptor expression and autophagy.

## Introduction

Chondrosarcoma denotes a heterogeneous group of neoplasms, comprised of tumor cells that share the common characteristic of producing extracellular matrix components in cartilage tissue [1]. With an incidence of 1:50,000 chondrosarcoma typically occurs in adults in their 3^rd^ to 6^th^ decade of life and represents the second most common primary malignant bone tumor in a large epidemiologic series [2]. Extensive surgical resection remains the best available treatment option for intermediate- to high-grade tumors as they are relatively chemo- and radiotherapy resistant, due to their extracellular matrix, low percentage of dividing cells, and poor vascularity [3, 4]. From the clinical point of view, preventing recurrence and finding better treatment options for unresectable or metastatic chondrosarcoma is a considerable challenge within the field of cancer treatment.

The ubiquitin proteasome pathway plays a significant part in the regulation of a variety of cellular processes dealing with the growth and survival of tumor cells. Generally it has been established that inhibition of proteasome activity not only leads to cell death but also induces cell autophagy [5, 6]. The role of autophagy in cancer cells is complex and context-dependent [7]. Some types of cancer cells may exploit autophagy to adapt to the hypoxic, nutrient limiting, and metabolically stressful tumor microenvironment, as well as therapeutically induced cell stress or damage [8]. On the other hand it can raise the efficiency of radiation therapy [9] and chemotherapy [10, 11] including the activity of inhibitors of histone deacetylase [12], hedgehog [13], and mTOR [14] respectively. It is therefore evident that therapeutically evoked autophagy improves the therapeutic efficiency of anti-cancer drugs [15]. Resistance to chemotherapy-induced apoptosis is one of the most important features of tumor cells, and also contributes to tumor recurrence and metastasis. There are significant indications that as a cell-protective mechanism, activation of the autophagy pathway plays an important role in apoptosis resistance [16].

Substances that inhibit the proteasome function could therefore function as anti-cancer agents and open up the search for new cancer therapies. In this context it has been previously demonstrated that the proteasome inhibitor bortezomib exhibits antitumor activity against a variety of malignancies. Bortezomib was the first proteasome inhibitor used in clinical practice and is now approved for the treatment of multiple myeloma [17]. Numerous clinical trials with bortezomib have shown its efficacy as an active antitumor agent against a variety of solid tumors such as colon cancer, prostate cancer, breast cancer, and ovarian cancer [18–20]. It has been applied as a single agent and in combination with other chemotherapeutic drugs, and showed potent effects. Clinical phase I and II studies using bortezomib in isolation or combined with other drugs have shown encouraging results in treating a variety of other hematological malignancies and solid tumors [21–26]. However, the effect of bortezomib on chondrosarcoma has not yet been investigated. Furthermore, due to the dual roles of autophagy in the survival and death of tumor cells, the effect of autophagy inhibition on human chondrosarcoma cells remains to be elucidated.

The aim of this study was to analyze the effect of the proteasome inhibitor bortezomib on cell growth and proliferation, as well as apoptosis and autophagy induction and the involvement of different signal transduction pathways in two human chondrosarcoma cell lines.

## Material and Methods

### Cell culture

Human chondrosarcoma cell lines SW-1353 (CLS, Eppelheim, Germany) and Cal-78 (DSMZ, Braunschweig, Germany) were cultured in Dulbecco’s-modified Eagle’s medium (DMEM-F12; GIBCO^®^, Invitrogen, Darmstadt, Germany), containing 5% fetal bovine serum (FBS), 1% L-glutamine, 100 units/ml Penicillin, 100 μg/ml Streptomycin, and 0.25 μg Amphotericin B (all GIBCO^®^, Invitrogen). Both cell lines were verified by short tandem repeat analysis using PowerPlex 16 System Kit (Promega, Mannheim, Germany). Cells were kept at 37°C in a humidified atmosphere of 5% CO_2_ and were passaged by trypsinization after reaching 80–90% confluence.

### Cell viability and proliferation assays

The MTS assay (Brand, Voerde-Friedrichsfeld, Germany) was used to measure the metabolic activity of cells: 5x10^3^ cells per well were seeded into 96 well plates and treated with 0–100 nM bortezomib (Selleckchem, Houston, TX). The cells were treated at 24, 48, and 72 h, after which a CellTiter 96 AQueous Assay (Promega, Madison, WI) was performed following the manufacturers' instructions; untreated cells were used as control. Data are the mean ± S.D. of three independent experiments each performed in quadruplicates. To analyze the effect of the lysosomal protease inhibitors on cell viability, cells were treated either with bortezomib alone or in combination with E64d or/and pepstatin A (each 10 μg/ml; both Sigma Aldrich, Vienna, Austria). Untreated cells were measured as controls (*n* = 8, mean ± S.D.).

The xCELLigence RTCA DP (ACEA Biosciences Inc., San Diego, CA) was used to monitor cell proliferation in real-time after cells were seeded on electronic microtiter plates (E-Plate; ACEA). Cells were treated with 0, 2.5, 5, and 10 nM bortezomib and the proliferation rate was measured at 96 h. Cell index (CI) measurements were performed in triplicate with a signal detection scheduled every 20 min. The cell index (CI) is a measure for the cell density and was normalized to the time point at which bortezomib was added. Following the continuous xCELLigence cell monitoring, the slope (1/h), representing the rate of change of the cell index, was calculated. The calculation was set within a 30 h time span and the start point was set 6 h after initiating the bortezomib treatment. Acquisition and analysis was performed with the RTCA software (Version 1.2, ACEA).

### Caspase-Glo^®^ 3/7 assay

1x10^4^ cells/well were treated with 5, 10, and 25 nM bortezomib for 1–72 h and analyzed for caspase 3/7 activation using the Caspase-Glo^®^ 3/7 Assay (Promega) according to the manufacturer´s protocol. Briefly, Caspase-Glo^®^ reagent was added 1:1 to sample volume into each well and incubated at room temperature for 30 min protected from light. Blank values were subtracted from the experimental values to exclude background luminescence and negative controls were used to determine basal caspase activity. Luminescence of each sample was measured using a Luminometer (LUMIstar; BMG Labtech, Ortenberg, Germany). Results were expressed as the mean from two independent experiments (*n* = 2, measured in biological quadruplicates) and error bars represent the S.D.

### Caspase-3 apoptosis assay

After incubation with 5 and 10 nM bortezomib for 48 h, cells were harvested by trypsinization, fixed with formaldehyde for 10 min at 37°C (2x10^6^ cells/ml), permeabilized with methanol, and re-suspended in incubation-buffer (FBS:PBS 1:200). Activation of caspase-3, a marker for cells undergoing apoptosis, requires proteolytic processing of its inactive zymogen into activated p17 and p12 fragments. The FITC-conjugated monoclonal cleaved caspase-3 (Asp175) antibody (Cell Signaling Technology, Danvers, MA) detects endogenous levels of the large fragment (17/19 kDa) of activated caspase-3 resulting from cleavage adjacent to aspartic acid175. The antibody does not recognize full length caspase-3 or other cleaved caspases. Cells were analyzed by flow cytometry (FACSCalibur™, BD Biosciences, San Jose, CA) performed with FACSDiva software. Histograms were created using FCS3 express software (De Novo software, Los Angeles, CA). Untreated cells were used as negative control.

### Annexin V/PI apoptosis assay

The APC Annexin V Apoptosis Detection Kit (BioLegends, San Diego, CA) was performed following the manufacturers´ instructions. Apoptotic cells were identified by incubation of 1x10^5^ cells in 100 μl Annexin V Binding buffer containing 5 μl Annexin V-APC and 5 μl PI for 15 min at room temperature. Flow cytometry analysis was performed with FACSCalibur™ (BD Biosciences). 10,000 events were collected. Cells were identified by side scatter and forward scatter with linear scale. Compensation was performed by single Annexin and PI measurements and analyzed by FCS3 express software (De Novo software). Untreated cells were used as negative control.

### Protein array

The Human Apoptosis Antibody Array (Abcam, Cambridge, UK) is suitable for the multiplex protein detection of 43 human apoptotic markers. Cells were treated with the respective IC_50_ value of bortezomib for 24 h and whole cell protein extracts were prepared with lysis buffer. Capture and control antibodies of the major contributors to apoptosis were spotted in duplicate on nitrocellulose membranes. The array was performed following the manufacturers' instructions; untreated cells were used for control.

### Reverse transcription polymerase chain reaction (RT-PCR)

Total ribonucleic acid (RNA) was isolated from treated and untreated cells with the RNeasy Mini Kit and DNase-I treatment according to the manufacturer's manual (Qiagen, Hilden, Germany). One microgram RNA was reverse transcribed using a RevertAid cDNA Synthesis Kit (Thermo Fisher Scientific, Waltham, MA). Amplification was performed with the Platinum SYBR Green Super Mix with ROX (Invitrogen) and measured by the AB7900HT instrument (Applied Biosystems, Invitrogen). Each qPCR run consisted of a standard 3-step PCR temperature protocol (annealing temperature of 60°C) followed by a melting curve protocol to confirm a single gene-specific peak and to detect primer dimerization. Relative quantification of expression levels were obtained by the ΔΔCt method based on the geometric mean of the internal controls glyceraldehyde 3-phosphate dehydrogenase (GAPDH), β-actin (ACTB), and hypoxanthine phosphoribosyl-transferase (HPRT-1), respectively. The following QuantiTect primer assays (Qiagen) were used for real time RT-PCR: Bax, Bak, Bcl-2, Bcl-xl, Atg5, Atg7, Atg12, Beclin, IGF1R, Fas, TRAILR-1, and TRAILR-2. The expression level (C_T_) of the target gene was normalized to the reference genes (ΔC_t_), the ΔC_t_ of the test sample was normalized to the ΔC_t_ of the control (ΔΔC_t_). Finally, the expression ratio was calculated with the 2^-ΔΔCt^ method (* p < 0.05).

### Western blot analysis

For immunoblotting, whole cell protein extracts were prepared with lysis buffer (50 mM Tris-HCl pH 7.4, 150 mM NaCl, 1 mM NaF, 1 mM EDTA, 1% NP-40, 1mM Na3 VO4, and protease inhibitor cocktail (P8340; Sigma Aldrich), subjected to SDS-PAGE and blotted onto Amersham™ Protran™ Premium 0.45 μM nitrocellulose membrane (GE healthcare Life science, Little Chalfont, UK). All steps were performed on ice. Protein concentration was determined with the Pierce BCA Protein Assay Kit (Thermo Fisher Scientific) according to the manufacturer's protocol. Primary antibodies against Atg5/12, Beclin, cytochrome C, and β-actin were purchased from Santa Cruz (Santa Cruz Biotechnology, Santa Cruz, CA). Fas, TNF-R1, TNF-R2, TRADD, and RIP antibodies were purchased from Cell Signaling Technology (Cell Signaling Technology). For cytochrome C immunoblotting cells were incubated with 0, 2.5, and 5 nM bortezomib for 24 h. Nuclear and cytoplasmic protein extracts were isolated according to the manufacturer's protocol (Active Motif North America, Carlsbad, CA). Subsequently, cells were collected in ice-cold PBS containing phosphatase inhibitors, centrifuged and re-suspended in hypotonic buffer to swell cell membranes. Detergent was added to lyse cell membranes and the suspension was centrifuged at 14,000 x g to separate the nuclei from the cytoplasmatic protein fraction. The cytoplasmic extracts were collected from the supernatant containing all cell organelles except the nuclei. After an additional centrifugation step, nuclei were lysed, and the nucleic extracts were collected in the presence of a protease inhibitor cocktail. For LC3B I-II immunoblotting cells were treated with the lysosomal protease inhibitors E64d and pepstatin A (10 μg/ml; both Sigma Aldrich), which were used to block autophagic flux and inhibit the degradation of LC3B-II [27]. Blots were developed using a horseradish peroxidase- conjugated secondary antibody (Dako, Jena, Germany) at room temperature for 1 h and the Amersham™ ECL™ prime western blotting detection reagent (GE Healthcare), in accordance with the manufacturer‘s protocol. Chemiluminescence signals were detected with the ChemiDocTouch Imaging System (BioRad Laboratories Inc., Herkules, CA) and images were processed with the ImageLab 5.2 Software (BioRad Laboratories Inc.).

### Immunofluorescence

5x10^5^ cells were seeded in polystyrene culture slides (BD Biosciences) and incubated either without, or with 5 nM (Cal-78) or 10 nM (SW-1353) bortezomib for 24 h. In addition, both cell lines where treated with the lysosomal protease inhibitors E64d and pepstatin A (10 μg/ml; both Sigma Aldrich) [27]. After incubation, cells were washed with PBS, fixed with 4% formaldehyde in PBS for 15 min at room temperature and washed with PBS. Subsequently, cells were permeabilized for 10 min at 4°C with ice-cold methanol. Cells were blocked 5 min at room temperature with UltraVision Protein Block (Thermo Fisher Scientific) and treated with the primary antibody against LC3B purchased from Cell Signaling Technology or rabbit-IgG (Linaris Biologische Produkte, Dossenheim, Germany) for negative controls overnight at 4°C. After an incubation of 2 h at room temperature with Alexa Fluor^®^ 488-conjugated AffiniPure Goat Anti-Rabbit IgG secondary antibody (Jackson Immunoresearch, Suffolk, UK), nuclei were counterstained and slides were mounted with Vectashield Mounting Medium with DAPI (Vector Laboratories, Burlingame, CA). Cells were stored in the dark and viewed at a 510 LSM Meta confocal microscope (Zeiss, Vienna, Austria) using excitation at 405 nm and detection with a BP 420–480 nm filter for the nuclear stain. Binding of the LC3B antibody was measured by excitation at a wavelength of 488 nm and detection with a LP 505 nm filter. ZEN 2009 Software was used to capture and process images.

### Statistical analysis

The outcome variables were expressed as mean ± SD. Student’s unpaired t-test and the exact Wilcoxon test were used to evaluate differences between groups with the PASW statistics 18 software (IBM Corporation, Somers, NY). Two-sided P-values below 0.05 were considered statistically significant. The significance of dose or time responses was assessed by repeated measures analysis. Graphical data were prepared and calculated with SigmaPlot^®^ (Systat Software Inc., San Jose, CA).

## Results

### Bortezomib reduces viability and cell proliferation of chondrosarcoma cells

**Fig 1:**
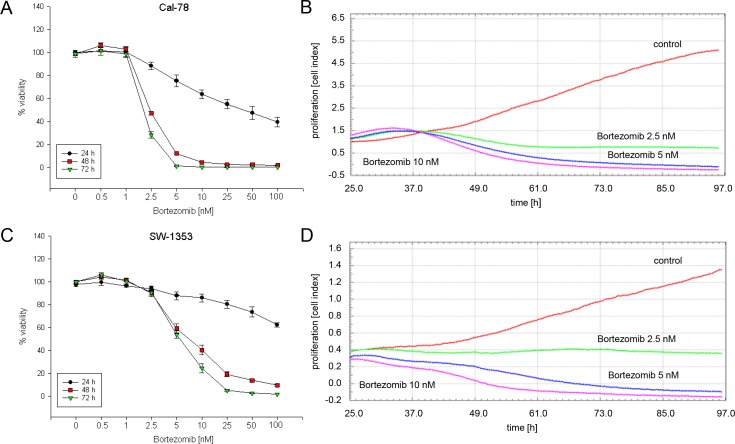
Influence of bortezomib on cell viability and proliferation of chondrosarcoma cells. A,C) After 24, 48, and 72 h incubation bortezomib inhibited cell growth in a concentration dependent manner. Untreated cells were measured as controls (*n* = 12, mean ± S.D.). B,D) Dynamic proliferation curves for Cal-78 and SW-1353 cells in the presence of 0 (red), 2.5 nM (green), 5 nM (blue), and 10 nM (purple) bortezomib. Data shown are representatives from three independent experiments (*n* = 3, measured in biological quadruplicates).

After treatment with 0–100 nM bortezomib for 24, 48, and 72 h, cells were measured by the MTS assay (n = 12). Bortezomib inhibited cell growth in a time- and dose-dependent manner in both cell lines (Fig 1A and 1C). The IC_50_ values after an incubation of 48 h were 2.36 nM for Cal-78 cells and 4.95 nM for SW-1353 cells. Cell growth curves were automatically recorded in real-time by the xCELLigence System over the entire time-period of 72 h and with three different concentrations of bortezomib (2.5 nM, 5 nM, 10 nM), (Fig 1B and 1D). In Cal-78 cells slope values changed under the influence of bortezomib from 0.015 ± 0.0003 (ctrl) to -0.025 ± 0.0002 (2.5 nM), -0.04 ± 0.0002 (5 nM) and -0.053 ± 0.0003, respectively. In SW-1353 cells slopes of 0.073± 0.0006 (ctrl), 0.019 ± 0.0012 (2.5 nM), -0.02 ± 0.0002 (5 nM) and -0.039 ± 0.00016 (10 nM) were measured.

### Bortezomib induces apoptosis through the mitochondria-caspase dependent pathway

**Fig 2:**
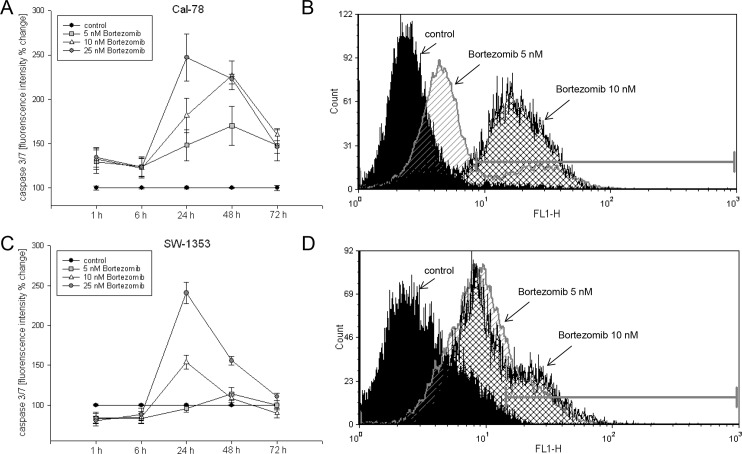
Apoptotic induction of bortezomib. A,C) Bortezomib treated chondrosarcoma cells showed a significantly higher level of caspase 3/7 activity than untreated cells. Untreated control cells served as reference value (ratio = 1). B,D) Cleavage of caspase-3 was detected after 24 h of bortezomib treatment by flow cytometry. The y-axis denotes cell counts and the x-axis represents fluorescence intensity of the APC antibody. The black filled histogram represents untreated control cells, the striated histogram represents 5 nM, and the checkered histogram shows 10 nM bortezomib treated cells.

To elucidate the apoptotic potential of bortezomib on chondrosarcoma cells, we performed a series of apoptosis assays. Apoptosis induction was investigated by quantitative measurement of the caspase 3/7 activity for a time period between 6 and 72 h and flow cytometric analysis (FACS) of caspase-3 cleavage after 24 h (Fig 2). Caspase 3/7 activity peaked after 24 h as a result of bortezomib treatment in Cal-78 and SW-1353 cells (Fig 2A and 2C). Accordingly, cleaved caspase-3 measurements performed after 24 h exposure to 5 and 10 nM bortezomib resulted in a significant increase of caspase-3 cleavage from 5.89±2.11% (ctrl) to 24.14±4.63% (5 nM; p < .05) and 69.31±7.78% (10 nM; p < .001) in Cal-78 cells and 3.83±1.67% (ctrl) to 32.45±4.23% (5 nM; p < .05) and 36.59±12.19% (10 nM; p < .05) in SW-1353 cells (n = 4) (Fig 2B and 2D). The FACS histograms represent untreated cells (black filled) versus 5 nM (striated lines) and 10 nM (checkered lines) bortezomib treated cells. In addition, apoptosis induction was investigated by Annexin V/PI staining (data not shown). Compared to untreated control cells, Cal-78 cells treated with 2.5 and 5 nM bortezomib showed a three to eightfold increase in Annexin positive cells after 24 h and a 10–18 fold increase after 48 h. Under the same conditions SW-1353 detailed a threefold increase in Annexin positive cells after 24 h and a fivefold increase after 48 h (*n* = 3).

**Fig 3:**
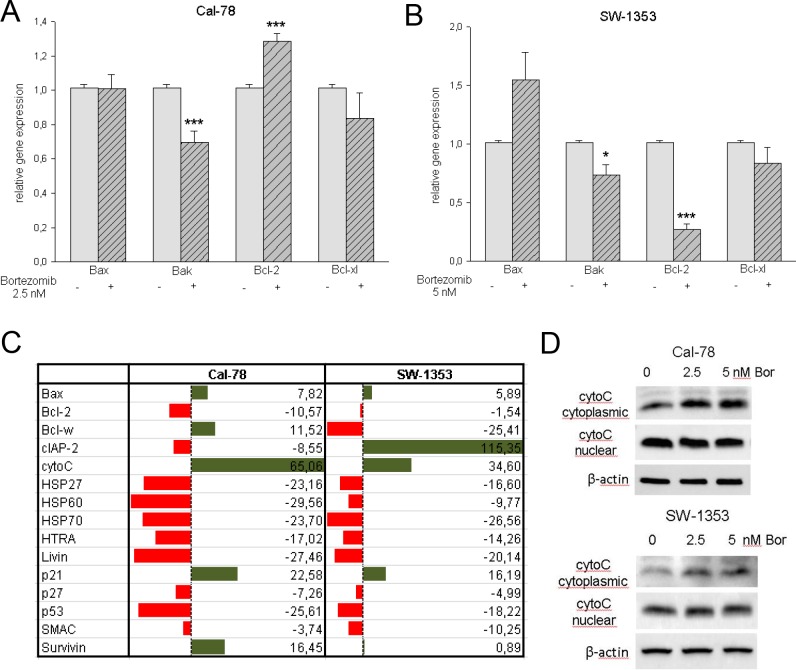
Analysis of the mitochondria-caspase dependent pathway. A,B) Relative gene expression analysis of the pro- and anti-apoptotic markers Bax, Bak, Bcl-2, and Bcl-xl in Cal-78 and SW-1353 cells treated with the respective IC_50_ values of bortezomib for 24 h (*n* = 10). C) The human apoptosis antibody protein array revealed the downregulation of the heat shock proteins HSP27, HSP60, and HSP70 as well as HTRA, Livin, p27, and p53 in both cell lines. D) Western blot analysis showed an upregulation of cytochrome C in the cytoplasmic fraction after bortezomib treatment.

In order to investigate the proteins that are responsible for the observed effect on apoptosis we performed RT-PCR and a human apoptosis antibody protein array for simultaneous detection of 43 human apoptotic markers (Fig 3). Relative mRNA expression levels of Bax, Bak, Bcl-2, and Bcl-xl were analysed by real time RT-PCR after treatment with the respective IC_50_ concentrations of bortezomib for 24 h. Untreated control cells served as a reference value (ratio = 1). In the case of Cal-78 cells, treatment with bortezomib induced significant downregulation of Bak (0.69±0.14; p < .001) and Bcl-2 (1.28±0.15; p < .001), whereas Bax and Bcl-xl remained unaffected (Fig 3A). Compared to untreated control cells SW-1353 cells showed an increase of Bax (1.55±0.72) and a significant decrease of the expression of Bak (0.74±0.20; p < .05) and Bcl-2 (0.27±0.13; p < .001) within the observation period (Fig 3B). Using the human apoptosis antibody protein array, the downregulation of the heat shock proteins HSP27, HSP60, and HSP70, HTRA, Livin, p27, and p53 was shown in both cell lines (Fig 3C). A particularly significant upregulation in the expression of cytochrome C was closely monitored. Western blot analysis of the cytoplasmic and nuclear protein extracts revealed an upregulation of cytochrome C in the cytoplasmic fraction after bortezomib treatment, whereas, the expression in the nuclear extract revealed no significant changes (Fig 3D).

### Bortezomib increased the expression of death receptors

**Fig 4:**
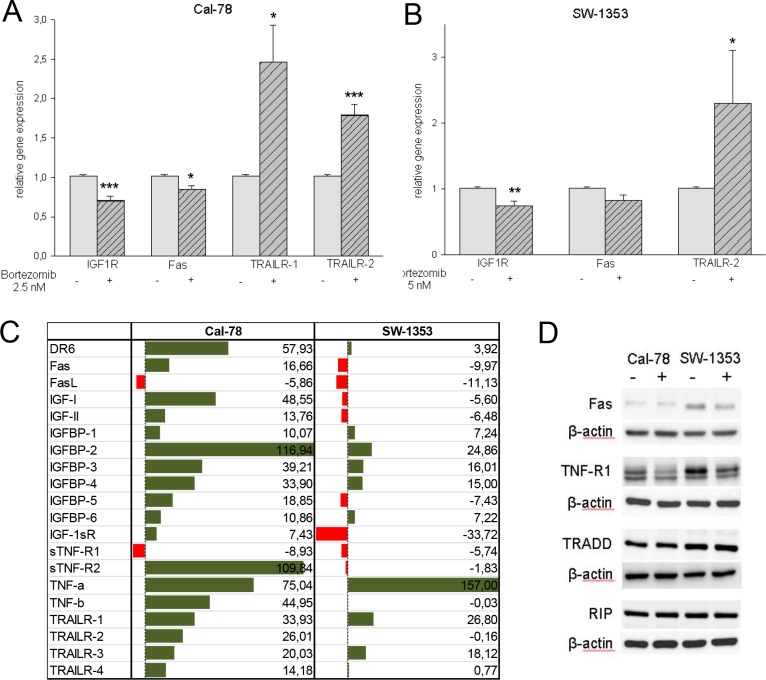
Analysis of TRAIL/death receptor pathway. A,B) Relative gene expression analysis of IGF1R, Fas, and the death receptors TRAILR-1 and TRAILR-2 after bortezomib treatment with the respective IC_50_ concentrations for 48 h. Untreated control cells served as reference value (ratio = 1). C) The human apoptosis antibody protein array supported the important role of the IGF binding proteins (IGFBP-1 to IGFBP-6), the TNF receptor family, and the TRAIL receptors in both cell lines. D) Western blot analysis showed the downregulation of Fas and TNF-R1.

To investigate the expression of insulin-like growth factor 1 receptor (IGF1R), the tumor necrosis factor (TNF)-related apoptosis-inducing ligand (TRAIL) receptors TRAIL-R1, TRAIL-R2, and Fas (CD95/Apo-1) on chondrosarcoma cells by bortezomib treatment, cells were incubated with the respective IC_50_ concentrations for 24 h. Relative mRNA expression levels were then analysed by real time RT-PCR. Untreated control cells served as a reference value (ratio = 1). Compared to untreated control cells both cell lines showed a significant decrease of IGFR1 (Cal-78: 0.66±0.14; p < .001 and SW-1353: 0.76±0.21; p < .01) and a decrease of the expression of Fas (Cal-78: 0.84±0.14; p < .05 and SW-1353: 0.82±0.24) within the observation period (Fig 4A and 4B). Furthermore, TRAILR-1 and TRAILR-2 were quantified by RT-PCR. Bortezomib treatment increased transcript expression of TRAILR-1 (Cal-78: 2.46±1.32; p < .05; SW-1353: not detectable) and TRAILR-2 (Cal-78: 1.79±0.39; p < .001 and SW-1353: 2.29±1.27; p < .05) significantly. The apoptosis antibody protein array confirmed all these results and supported the important role of the IGF binding proteins (IGFBP-1 to IGFBP-6), the TNF receptor family, and the TRAIL receptors (Fig 4C). In order to substantiate our observations, western blot analysis for the Fas, TNF-R1, TRADD, and RIP were performed (Fig 4D). Corresponding to the real time RT-PCR data, the expression of Fas and TNF-R1 were downregulated after treatment with the proteasome inhibitor. No changes were observed in the expression of TNFR-associated death domain (TRADD) and the receptor-interacting protein 1 (RIP).

### Bortezomib increased the expression of autophagy-associated proteins

**Fig 5:**
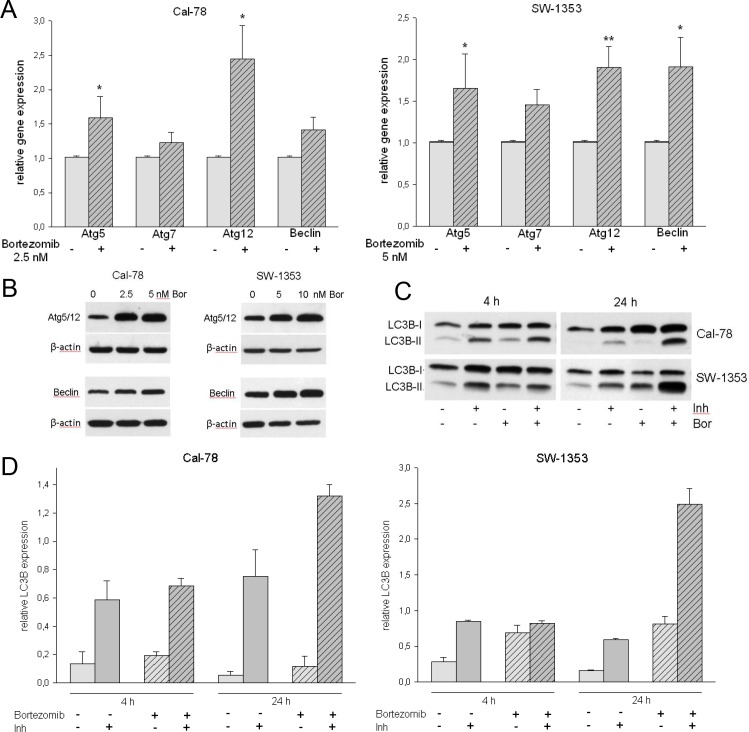
Bortezomib induces autophagy in human chondrosarcoma cells. A) Relative gene expression and B) western blot analysis of whole cell lysates for the expression of the autophagy markers Atg 5/12 and Beclin in Cal-78 and SW-1353 cells treated with the respective IC_50_ values of bortezomib for 24 h. C) Western blot analysis for the expression of LC3BI-II. The lysosomal protease inhibitors E64d and pepstatin A (Inh) blocked the autophagic flux and inhibited the degradation of LC3B-II. D) Quantification of the relative LC3B protein expression.

**Fig 6:**
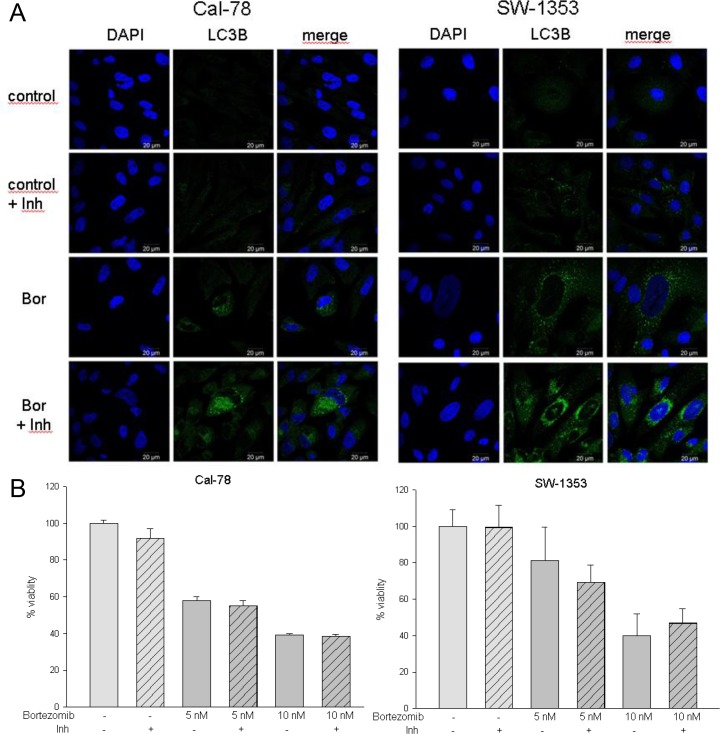
LC3B immunostaining. A) Effect of bortezomib treatment on LC3B immunostaining of Cal-78 and SW-1353 cells. Double immunolabeling with DAPI (blue) and anti-LC3B (green) antibodies was performed as described in the methods section. The lysosomal protease inhibitors E64d and pepstatin A (Inh) blocked the autophagic flux and inhibited the degradation of LC3B-II (bar: 20 μm). B) Cal-78 and SW-1353 cells were treated either with bortezomib alone or in combination with the lysosomal protease inhibitors E64d and pepstatin A (Inh; striated bars) and cell viability was analysed by the MTS assay. Untreated cells were measured as controls (light grey; *n* = 8, mean ± S.D.).

Studies have shown that autophagy plays a key role in tumor survival and apoptosis. Therefore, we monitored the expression of the autophagy-associated proteins Atg5/12, Beclin, and LC3I-II in chondrosarcoma cells following treatment with bortezomib. Both the relative gene expression and the protein expression analysis revealed a significant upregulation of Atg5, Atg12, and Beclin (Fig 5A and 5B). Bortezomib treatment increased transcript expression of Atg5 (Cal-78: 1.59±0.81; p < .05 and SW-1353: 1.66±1.09; p < .05), Atg7 (Cal-78: 1.23±0.42 and SW-1353: 1.46±0.52), Atg12 (Cal-78: 2.45±1.37; p < .05 and SW-1353: 1.91±0.69; p < .01), and Beclin (Cal-78: 1.41±0.54 and SW-1353: 1.92±0.91; p < .05) significantly. Western blot data (Fig 5B) confirmed that bortezomib increased the protein expression of the autophagy related proteins Atg5/12 and Beclin in both chondrosarcoma cell lines. When autophagy occurs, the microtubule-associated protein LC3B localizes to isolation membranes leading to the formation of autophagosome membranes. LC3B-I, the cellular form, converts to LC3B-II when autophagy happens and the amount of LC3B-II becomes a marker for the formation of autophagosomes. As shown in Fig 5C bortezomib apparently increased the expressions of LC3B-II. The quantification of the relative LC3B protein expression is shown in Fig 5D. The change in the subcellular distribution of LC3B can also be followed by immunofluorescence microscopy. The characteristic pattern of LC3B puncta can be observed in autophagic cells stained with anti-LC3B antibody, whereas untreated control cells revealed only a small quantity of LC3B positivity (Fig 6). The effect of the lysosomal protease inhibitors E64d and pepstatin A can be easily observed. Inhibition of the autophagic flux led to prevention of LC3B‑II degradation, which subsequently resulted in LC3B‑II accumulation (Fig 6A, last row). MTS experiments with both chondrosarcoma cell lines treated either with bortezomib alone or in combination with the lysosomal protease inhibitors E64d and pepstatin A (each 10 μg/ml), indicated that these inhibitors did not exhibit significant effects on cell viability (Fig 6B).

## Discussion

The observation that cell death pathways are often disabled in cancer cells has been an important motivation to target these pathways as an anti-cancer strategy. To develop new cancer treatment options, a fundamental requirement is an understanding of the interrelationship between different cell death pathways, as well as between cell death and non-cell death pathways. Chondrosarcoma is characterized by its lack of response to conventional cytotoxic chemotherapy, propensity for developing lung metastases, and poor patient survival. Therefore, research in the field of cell death mechanisms is of particular importance. In the diverse group of human sarcomas, the effect of the proteasome inhibitor bortezomib has only been tested on liposarcoma cells [28]. In the present study, we have demonstrated for the first time that bortezomib reduced the cell viability and proliferation of human chondrosarcoma cells in a time- and dose-dependent manner, with IC_50_ values located in a low nM range. The underlying mechanism of the effect of bortezominb was examined in a series of apoptosis arrays, the expression of death receptor proteins, and the induction of autophagy in Cal-78 and SW-1353 chondrosarcoma cell lines.

Two pathways characterize apoptotic processes, both mediated by a family of cysteine proteases known as caspases: the intrinsic or mitochondrial pathway and the extrinsic or death receptor pathway. The intrinsic pathway involves the activation of pro-apoptotic molecules upon receiving intracellular stress signals. These molecules converge on the mitochondria to trigger the release of mitochondrial apoptogenic molecules under the control of the Bcl (B-cell lymphocytic-leukaemia proto-oncogene) proteins, a family which includes the anti-apoptotic members Bcl-2 and Bcl-xL, and the pro-apoptotic members Bax and Bak [29]. Bortezomib treatment resulted in a time-dependent increase of cleaved caspase-3 activity in Cal-78 and SW-1353 cells. In these two cell lines the caspase 3/7 activity peaked after 24 h. The induction of apoptosis in chondrosarcoma cells was further confirmed by cleaved caspase 3 FACS analysis and AnnexinV/PI staining. Relative mRNA expression levels revealed a significant downregulation of the pro-apoptotic marker Bax and a downregulation of the anti-apoptotic marker Bcl-2. In addition, the human apoptosis antibody protein array still showed the importance of the heat shock proteins (HSP) HSP27, HSP60, and HSP70. HSP’s protective function allows the cells to survive under lethal conditions. It is important to mention, that a diminished expression of HSP27, HSP60, and HSP70 increases the cells’ sensitivity to apoptotic stimuli [30, 31]. Within the intrinsic mitochondria-caspase dependent pathway, one of the released mitochondrial molecules is cytochrome C, which interacts with cytosolic apoptotic protease activation factor-1 (Apaf-1) and pro-caspase-9 to form the apoptosome, the caspase-3 activation complex [32]. A particularly significant upregulation in the expression of cytochrome C was observed in chondrosarcoma cells.

Tumor necrosis factor-related apoptosis-inducing ligand (TRAIL) triggers programmed cell death in various types of cancer cells without causing toxicity to normal cells [33]. Binding of TRAIL to the death receptors (TRAILR-1 and TRAILR-2) is essential for directing apoptosis in many types of tumor cells [34]. Within this context, death receptors contribute differentially to apoptotic signaling, depending tumor type. It has been reported that bortezomib sensitizes melanoma tumors to TRAIL-mediated apoptosis [28]. Bortezomib upregulated TRAILR-2 but not TRAILR-1 receptors cell surface expression in lung cancer cell lines [35] and significantly increased the transcript expression of TRAILR-1, TRAILR-2 and Fas in colorectal tumor cells [36]. Corresponding to the literature TRAILR-1, TRAILR-2, and DR6 were significantly upregulated in chondrosarcoma cells after bortezomib treatment. During cancer progression, the interaction between Fas and Fas-ligand (FasL) is largely impaired due to suppression of Fas expression on tumor cells [37]. Together these results suggested that bortezomib enhances TRAIL sensitivity in human chondrosarcoma cells through regulating expression of death receptors.

The role of autophagy as an alternative cell death mechanism has in recent years been a topic of debate. *In vivo*, chemotherapy and radiotherapy are known to induce both cell death and autophagy in tumor cells and autophagy has been observed to confer a tumor suppressive effect in various animal models. Two ubiquitin-like conjugation systems are important for autophagosome elongation. The first one involves Atg5 and Atg12 proteins and the other requires the conjugation of microtubule-associated protein 1 light chain 3 (LC3B). The covalent attachment of Atg12 to Atg5 is mediated by Atg7 [38]. Our data demonstrates the significant increase of the relative mRNA expression levels and the protein levels of the autophagy marker Atg 5, Atg 12, and Atg 7. In mammals, the autophagic process undergoes regulation by Beclin1, a protein able to promote autophagy and inhibit tumorigenesis [39]. Beclin is upregulated in chondrosarcoma cell lines as well. When autophagy occurs, the microtubule-associated protein LC3B localizes to membranes leading to the formation of autophagosome membranes and the amount of LC3B-II becomes a marker for the formation of autophagosomes. If cells are treated with lysosomal protease inhibitors such as E64d and pepstatin A, degradation of LC3B‑II is partially inhibited, whereas that of LC3B‑I is not affected [27]. Autophagy inhibitors were used in this work to improve the quality of Western blot images. Although these experiments showed that the use of the autophagy inhibitors did not modulate bortezomib-induced apoptosis during *in vitro* experiments, this was not the primary research focus.

As shown in Fig 5C and 5D and Fig 6 bortezomib apparently increased the expressions of LC3B-II. These results clearly indicate that bortezomib can induce autophagy in human chondrosarcoma cells.

In any case, the in vivo functional relationship between apoptosis ('self-killing') and autophagy ('self-eating') is complex given that under certain circumstances autophagy constitutes a stress adaptation to avoid cell death (thereby suppressing apoptosis). In contrast, in other cellular conditions autophagy provides an alternative cell-death pathway. Autophagy and apoptosis may be triggered by common upstream signals, which can result in a combined autophagy and apoptosis response. Alternatively, the cell may switch between the two responses in a mutually exclusive manner. On a molecular level, this means that the apoptotic and autophagic response machineries share common pathways that can either link or polarize the cellular responses. Depending on the physiopathological setting, it has been proposed that autophagy could protect the cell from apoptosis. Conversely, autophagy may act as an alternative pathway to apoptosis to induce cell death. Finally, autophagy could also act together with apoptosis as a combined mechanism for cell death [40].

Our results demonstrate for the first time that bortezomib decreased the viability of human chondrosarcoma cells and induced apoptosis through the mitochondrial-caspase dependent pathway and death receptor regulation. In addition, bortezomib increased autophagy. In light of the complex interplay between different cell death and autophagic pathways, it is important to consider the potential interactions and complications that may arise when administering cell death- and autophagy-regulating therapies for cancer treatment.

In summary, our findings strongly support bortezomib as an interesting target for further investigation and development of novel therapeutics in sarcoma research.

## References

[1] Fletcher CDM, Hogendoorn PCW, Mertens F. Chondrosarcoma (grades I-III) including primary and secondary variants and periosteal chondrosarcoma In: World Health Organization Classification of Tumours of Soft Tissue and Bone 4^th^ edn Geneva: IARC Press; 2013 pp.264–74.

[2] Damron TA, Ward WG, Stewart A. Osteosarcoma, chondrosarcoma, and Ewing's sarcoma. National Cancer Data Base Report. Clin Orthop Relat Res 2007;459:40–47. 10.1097/BLO.0b013e318059b8c9 17414166

[3] Giuffrida AY, Burgueno JE, Koniaris LG, Gutierrez JC, Duncan R, Scully SP. Chondrosarcoma in the United States (1973 to 2003): an analysis of 2890 cases from the SEER database. J Bone Joint Surg Am 2009; 91(5):1063–1072. 10.2106/JBJS.H.00416 19411454

[4] van Maldegem AM, Gelderblom H, Palmerini E, Dijkstra SD, Gambarotti M, Ruggieri P, et al Outcome of advanced, unresectable conventional central chondrosarcoma. Cancer 2014;120(20):3159–3164. 10.1002/cncr.28845 24995550

[5] Dalby KN, Tekedereli I, Lopez-Berestein G, Ozpolat B. Targeting the prodeath and prosurvival functions of autophagy as novel therapeutic strategies in cancer. Autophagy 2010;(3):322–329. 20224296 10.4161/auto.6.3.11625 PMC2914492

[6] Long JS, Ryan KM. New frontiers in promoting tumour cell death: targeting apoptosis, necroptosis and autophagy. Oncogene 2012;31:5045–5060. 10.1038/onc.2012.7 22310284

[7] White E. Deconvoluting the context-dependent role for autophagy in cancer. Nat Rev Cancer 2012;12:401–410. 10.1038/nrc3262 22534666 PMC3664381

[8] Rouschop KM, Wouters BG. Regulation of autophagy through multiple independent hypoxic signaling pathways. Curr Mol Med 2009;9:417–424. 19519399 10.2174/156652409788167131

[9] Apel A, Herr I, Schwarz H, Rodemann HP, Mayer A. Blocked autophagy sensitizes resistant carcinoma cells to radiation therapy. Cancer Res 2008;68:1485–1494. 10.1158/0008-5472.CAN-07-0562 18316613

[10] Qadir MA, Kwok B, Dragowska WH, To KH, Le D, Bally MB, et al Macroautophagy inhibition sensitizes tamoxifen-resistant breast cancer cells and enhances mitochondrial depolarization. Breast Cancer Res Treat 2008;112:389–403. 10.1007/s10549-007-9873-4 18172760

[11] Amaravadi RK, Yu D, Lum JJ, Bui T, Christophorou MA, Evan GI, et al Autophagy inhibition enhances therapy-induced apoptosis in a Myc-induced model of lymphoma. J Clin Invest 2007;117:326–336. 10.1172/JCI28833 17235397 PMC1765515

[12] Carew JS, Nawrocki ST, Kahue CN, Zhang H, Yang C, Chung L, et al Targeting autophagy augments the anticancer activity of the histone deacetylase inhibitor SAHA to overcome Bcr-Abl-mediated drug resistance. Blood 2007;110:313–322. 10.1182/blood-2006-10-050260 17363733 PMC1896119

[13] Ridzewski R, Rettberg D, Dittmann K, Cuvelier N, Fulda S, Hahn H. Hedgehog Inhibitors in Rhabdomyosarcoma: A Comparison of Four Compounds and Responsiveness of Four Cell Lines. Front Oncol 2015;5:130–140. 10.3389/fonc.2015.00130 26106586 PMC4459089

[14] Wong PM, Feng Y, Wang J, Shi R, Jiang X. Regulation of autophagy by coordinated action of mTORC1 and protein phosphatase 2A. Nat Commun 2015;6:8048–8059. 10.1038/ncomms9048 26310906 PMC4552084

[15] Sui X, Chen R, Wang Z, Huang Z, Kong N, Zhang M, et al Autophagy and chemotherapy resistance: a promising therapeutic target for cancer treatment. Cell Death Dis 2013;4(10):e838. 24113172 10.1038/cddis.2013.350 PMC3824660

[16] El-Khattouti A, Selimovic D, Haikel Y, Hassan M. Crosstalk Between Apoptosis and Autophagy: Molecular Mechanisms and Therapeutic Strategies in Cancer. J Cell Death 2013;6:37–55. 10.4137/JCD.S11034 25278778 PMC4147769

[17] Frankland-Searby S, Bhaumik SR. The 26S proteasome complex: an attractive target for cancer therapy. Biochem Biophys Acta 2012;1825:64–76. 10.1016/j.bbcan.2011.10.003 22037302 PMC3242858

[18] Ludwig H, Khayat D, Giaccone G, Facon T. Proteasome inhibition and its clinical prospects in the treatment of hematologic and solid malignancies. Cancer 2005;104:1794–1807. 10.1002/cncr.21414 16178003

[19] Muñoz-Galván S, Gutierrez G, Perez M, Carnero A. MAP17 (PDZKIP1) Expression Determines Sensitivity to the Proteasomal Inhibitor Bortezomib by Preventing Cytoprotective Autophagy and NFκB Activation in Breast Cancer. Mol Cancer Ther 2015;14(6):1454–1465. 10.1158/1535-7163.MCT-14-1053 25837675

[20] Kao C, Chao A, Tsai CL, Chuang WC, Huang WP, Chen GC et al Bortezomib enhances cancer cell death by blocking the autophagic flux through stimulating ERK phosphorylation. Cell Death Dis 2014;5:e1510 10.1038/cddis.2014.468 25375375 PMC4260726

[21] Blaney SM, Bernstein M, Neville K, Ginsberg J, Kitchen B, Horton T, et al Phase I study of the proteasome inhibitor bortezomib in pediatric patients with refractory solid tumors: a Children’s Oncology Group study (ADVL0015). J Clin Oncol 2004;22:4804–4809. 10.1200/JCO.2004.12.185 15570082

[22] Messersmith WA, Baker SD, Lassiter L, Sullivan RA, Dinh K, Almuete VI, et al Phase I trial of bortezomib in combination with docetaxel in patients with advanced solid tumors. Clin Cancer Res 2006;12:1270–1275. 10.1158/1078-0432.CCR-05-1942 16489083

[23] Piperdi B, Walsh WV, Bradley K, Zhou Z, Bathini V, Hanrahan-Boshes M, et al Phase-I/II study of bortezomib in combination with carboplatin and bevacizumab as first-line therapy in patients with advanced non-small-cell lung cancer. J Thorac Oncol 2012;7:1032–1040. 10.1097/JTO.0b013e31824de2fa 22534815 PMC3852685

[24] Besse B, Planchard D, Veillard AS, Taillade L, Khayat D, Ducourtieux M, et al Phase 2 study of frontline bortezomib in patients with advanced non-small cell lung cancer. Lung Cancer 2012;76:78–83. 10.1016/j.lungcan.2011.09.006 22186627

[25] Poklepovic A, Youssefian LE, Winning M, Birdsell CA, Crosby NA, Ramakrishnan V, et al Phase I trial of bortezomib and dacarbazine in melanoma and soft tissue sarcoma. Invest New Drugs 2013;31(4):937–942. 10.1007/s10637-012-9913-8 23315028 PMC3844155

[26] Fenichel MP. FDA approves new agent for multiple myeloma. J Natl Cancer Inst 2015;107(6):djv165 10.1093/jnci/djv165 26032612

[27] Mizushima N, Yoshimori T. How to interpret LC3 immunoblotting. Autophagy 2007;3:542–545. 17611390 10.4161/auto.4600

[28] Hu Y, Wang L, Wang L, Wu X, Wu X, Gu Y, et al Preferential cytotoxicity of bortezomib toward highly malignant human liposarcoma cells via suppression of MDR1 expression and function. Toxicol Appl Pharmacol 2015;283(1):1–8. 10.1016/j.taap.2014.12.015 25576094

[29] Wei MC, Zong WX, Cheng EH, Lindsten T, Panoutsakopoulou V, Ross AJ, et al Proapoptotic BAX and BAK: a requisite gateway to mitochondrial dysfunction and death. Science 2001;92:727–730. 10.1126/science.1059108 PMC3049805 11326099

[30] Kamada M, So A, Muramaki M, Rocchi P, Beraldi E, Gleave M. Hsp27 knockdown using nucleotidebased therapies inhibit tumor growth and enhance chemotherapy in human bladder cancer cells. Mol Cancer Ther 2007;6:299–308. 10.1158/1535-7163.MCT-06-0417 17218637

[31] Lanneau D, Brunet M, Frisan E, Solary E, Fontenay M, Garrido C. Heat shock proteins: essential proteins for apoptosis regulation. J Cell Mol Med 2008;12(3):743–761. 10.1111/j.1582-4934.2008.00273.x 18266962 PMC4401125

[32] Li P, Nijhawan D, Budihardjo I, Srinivasula SM, Ahmad M, Alnemri ES, et al Cytochrome c and dATP-dependent formation of Apaf-1/caspase-9 complex initiates an apoptotic protease cascade. Cell 1997;91:479–489. 9390557 10.1016/s0092-8674(00)80434-1

[33] Ashkenazi A, Pai RC, Fong S, Leung S, Lawrence DA, Marsters SA, et al Safety and antitumor activity of recombinant soluble Apo2 ligand. J Clin Invest 1999;104:155–162. 10.1172/JCI6926 10411544 PMC408479

[34] LeBlanc HN, Ashkenazi A. Apo2L/TRAIL and its death and decoyreceptors. Cell Death Differ 2003;10:66–75. 10.1038/sj.cdd.4401187 12655296

[35] Luster TA, Carrell JA, McCormick K, Sun D, Humphreys R. Mapatumumab and lexatumumab induce apoptosis in TRAIL-R1 and TRAIL-R2 antibody-resistant NSCLC cell lines when treated in combination with bortezomib. Mol Cancer Ther 2009;8:292–302. 10.1158/1535-7163.MCT-08-0918 19174554

[36] Cacan E, Spring AM, Kumari A, Greer SF, Garnett-Benson C. Combination Treatment with Sublethal Ionizing Radiation and the Proteasome Inhibitor, Bortezomib, Enhances Death-Receptor Mediated Apoptosis and Anti-Tumor Immune Attack. Int J Mol Sci 2015;16(12):30405–30421. 10.3390/ijms161226238 26703577 PMC4691179

[37] Pryczynicz A, Guzinska-Ustymowicz K, Kemona A. Fas/FasL expression in colorectal cancer. An immunohistochemical study. Folia Histochem Cytobiol 2010;48:425–429. 10.2478/v10042-010-0058-3 21071349

[38] Geng J, Klionsky DJ. Quantitative regulation of vesicle formation in yeast nonspecific autophagy. Autophagy 2008;4:955–957. 18758231 10.4161/auto.6791

[39] Liang XH, Jackson S, Seaman M, Brown K, Kempkes B, Hibshoosh H, et al Induction of autophagy and inhibition of tumorigenesis by beclin 1. Nature 1999;402:672–676. 10.1038/45257 10604474

[40] Maiuri MC, Zalckvar E, Kimchi A, Kroemer G. Self-eating and self-killing: crosstalk between autophagy and apoptosis. Nat Rev 2007; 8:741–752. 10.1038/nrm2239 17717517

